# Causes and Factors Affecting Cesarean Hysterectomy: A Retrospective Study

**DOI:** 10.3390/medicina61030371

**Published:** 2025-02-20

**Authors:** Ghazal Mansouri, Fatemeh Karami Robati, Azam Dehghani, Faezeh Golnarges, Hamid Salehiniya, Ibrahim Alkatout, Leila Allahqoli

**Affiliations:** 1 Department of Obstetrics and Gynecology, School of Medicine, Afzalipour Hospital, Kerman University of Medical Sciences, Kerman 76169-13555, Iran; dr_mansouri93@yahoo.com; 2 Clinical Research Development Unit, Afzalipour Hospital, Kerman University of Medical Sciences, Kerman 7616913555, Iran; fatemehk19@gmail.com (F.K.R.);; 3 Social Determinants of Health Research Center, Birjand University of Medical Sciences, Birjand 9717853577, Iran; 4 Department of Obstetrics and Gynecology, University Hospital Schleswig-Holstein, Campus Kiel, 24105 Kiel, Germany; ibrahim.alkatout@uksh.de; 5 Ministry of Health, Treatment and Medical Education, Tehran 1419943471, Iran; lallahqoli@gmail.com

**Keywords:** cesarean hysterectomy, complications, repeated cesarean sections, placenta accreta

## Abstract

*Background and Objectives*: Cesarean hysterectomy is a critical intervention often required to manage life-threatening postpartum hemorrhage (PPH) due to complications such as uterine atony, abnormal placental implantation, or traumatic rupture. Although lifesaving, the procedure is associated with significant risks and complications. This study investigates the causes and outcomes of cesarean hysterectomy, focusing on complications arising from the procedure. *Materials and Methods*: A retrospective analysis was conducted on 82 women who underwent cesarean hysterectomy at Afzali Pour Hospital between 2018 and 2022. All patients were followed for 42 days post-surgery to evaluate outcomes and complications. Data were extracted from electronic medical records, encompassing demographic, obstetric, and clinical details, including age, body mass index, previous cesarean sections, indications for cesarean deliveries, causes of hysterectomy, and complications. The primary outcome was to determine the causes of cesarean hysterectomy, while the secondary outcome assessed the complications associated with the procedure. Stepwise logistic regression analysis was utilized to identify significant predictors of complications. *Results*: The study included 82 women who underwent cesarean hysterectomy. The mean age of the participants was 35.2 years (SD = 5.4), with a range from 24 to 48 years. The average BMI was 29.1 kg/m^2^ (SD = 4.3), with 45% of the women classified as overweight or obese (BMI ≥ 25). The majority of the patients (70%) had a history of two or more previous cesarean sections, and the most common indication for cesarean hysterectomy was abnormal placentation, including placenta accreta (58%). Uterine rupture was reported in 13% of the cases. In terms of complications, bladder injury was the most common, occurring in 33.33% of women, followed by fever (20%), ureteral injury (13.33%), and hematoma (8.89%). Stepwise logistic regression analysis revealed that higher BMI significantly increased the odds of the outcome (OR = 4.18, 95% CI: 1.66–10.51, *p* = 0.002), and the number of previous cesarean sections was also a significant predictor (OR = 2.30, 95% CI: 1.17–4.53, *p* = 0.016). *Conclusions*: Placenta accreta and previa were the most frequent causes of cesarean hysterectomy, with bladder injury and fever being the most common complications. A higher number of previous cesareans and higher BMI significantly increase the likelihood of complications. Understanding these risk factors can improve patient management and surgical outcomes, highlighting the importance of careful monitoring and preoperative planning in women with a history of cesarean deliveries.

## 1. Introduction

Cesarean hysterectomy is a critical surgical intervention most commonly performed to manage life-threatening postpartum hemorrhage (PPH) caused by resistant uterine atony, traumatic rupture of the birth canal, or abnormal placental implantation [1]. This procedure is typically conducted during or immediately following a cesarean section when such severe complications arise [2]. According to the American College of Obstetricians and Gynecologists, cesarean hysterectomy is considered the gold standard for the definitive management of these conditions [3]. Secondary PPH, resulting from various etiologies, is also a leading indication for emergency hysterectomy during childbirth [1]. Although lifesaving, cesarean hysterectomy carries inherent risks, including significant bleeding, injury to the urinary and gastrointestinal systems, and an increased likelihood of postoperative complications such as infection, thrombosis, and pelvic injury [4]. The nature of the procedure—whether elective or emergent—plays a critical role in determining the risk of these complications [4]. While hysterectomy may sometimes be anticipated in cases of abnormal placental implantation, it remains a complex and high-risk procedure with a spectrum of potential complications that are often challenging to predict or prevent [2,3,5]. This study aims to explore the causes and factors influencing the outcomes of cesarean hysterectomy, with a particular focus on complications occurring during and after the procedure. Understanding these factors is crucial for optimizing surgical outcomes, minimizing complications, and improving patient care in obstetric practice.

## 2. Methods

### 2.1. Study Design and Patients

This retrospective study aimed to investigate the causes and complications associated with cesarean hysterectomy at Afzali Pour Hospital, a tertiary care center in Kerman, Iran, in 2024. The study included all women who underwent cesarean hysterectomy at this hospital between 2018 and 2022, utilizing a census method for participant selection. The inclusion criterion was restricted to patients who underwent hysterectomy during the same hospitalization following cesarean delivery. Patients with more than 10% missing data and those with a history of surgery, except for cesarean section, were excluded from the study. All hysterectomy procedures were performed via laparotomy by a consistent surgical team. Prior to the induction of general anesthesia for hysterectomy, spinal or epidural anesthesia was administered for cesarean delivery. Postoperatively, all patients were transferred to the intensive care unit (ICU) for initial postoperative management.

Data were extracted from electronic medical records, with the principal investigator accessing the medical records department of Afzali Pour Hospital to compile participant information using a structured data collection form. The data collection tool was validated and approved by a faculty member of the obstetrics and gynecology department. The tool comprised four sections, as outlined below:

**Patient Characteristics and Obstetrics History**

This section included demographic and clinical data such as age (years), gravidity, parity, gestational age (weeks), number of prior cesarean deliveries, indications for previous and current cesarean sections, and body mass index (BMI, kg/m^2^). BMI was categorized as follows: <25 (normal), 25–30 (overweight), and >30 (obese). Additionally, the American Society of Anesthesiologists (ASA) physical status classification was recorded.

**Causes of Hysterectomy and Hospitalization**

The primary causes of hysterectomy were documented, including uterine atony, placenta accreta, placenta previa, placenta increta, and placenta percreta. The mean duration of hospitalization (in days) was also recorded.

**Complications of Cesarean Hysterectomy**

Intra- and post-operative complications were assessed, including bladder injury, bowel injury, fever, hematoma, surgical site infections (SSIs), abdominal reoperation, pulmonary embolism (PE), ureteral injury, and fistula formation.

**Follow-Up and Readmission**

Patient status at discharge and reasons for readmission were reviewed. All participants were followed for 42 days post-discharge, with routine evaluations conducted every 2 weeks at the hospital’s obstetrics and gynecology clinic.

Fever was defined as an axillary temperature of ≥37.5 °C recorded within the first 48 h postoperatively. Intra-intestinal, fistula, and urogenital (ureter and bladder) injuries, as well as perioperative PE, were identified using appropriate ICD-10-CM codes, selected based on the injury’s nature, location, and classification as either accidental or a procedural complication. Hematoma was characterized as a localized accumulation of blood in or around the surgical site resulting from bleeding during or after the procedure, presenting as swelling, discoloration, or a palpable mass accompanied by pain, tenderness, or signs of infection. Infection was defined by the presence of clinical signs of inflammation caused by pathogenic microorganisms, confirmed by an elevated white blood cell count (>10,000/mm^3^ or above laboratory reference values), along with localized signs such as redness, warmth, swelling, pain, or purulent discharge, and systemic manifestations such as fever or positive microbial cultures. Reopening of the surgical site was defined as the separation or dehiscence of the wound occurring within 42 days postoperatively, involving partial or complete disruption of the incision with potential exposure of underlying tissues or organs, often associated with symptoms such as pain, redness, or discharge. All abdominal reoperations (relaparotomies) were included in the analysis, encompassing cases in which patients underwent reoperation prior to hospital discharge.

### 2.2. Study Outcome

The primary outcome of this study was to evaluate the causes of hysterectomy in women undergoing cesarean delivery. The secondary outcome focused on investigating the complications associated with cesarean hysterectomy, particularly in relation to factors such as age, BMI, number of prior cesarean deliveries, gravidity, parity, indications for the first and most recent cesarean sections, and the underlying causes of hysterectomy.

### 2.3. Statistical Analysis

Data analysis was conducted using SPSS version 25, with a significance threshold set at *p* < 0.05. Descriptive statistics, including frequencies, percentages, means, and standard deviations (SDs), were used to summarize both qualitative and quantitative data. The distribution of variables was assessed to determine the appropriate use of parametric or non-parametric statistical methods. To evaluate the relationships between independent variables and the outcome, logistic regression analysis was performed. This analysis incorporated all relevant variables, including patient age, BMI, number of previous cesareans, gravidity, parity, causes of both the first and recent cesareans, as well as the causes leading to hysterectomy, to better understand their impact on the incidence of complications. A backward conditional approach was applied to identify significant predictors of the outcome. The final model included variables with statistically significant associations, and odds ratios (ORs) with 95% confidence intervals (CIs) were reported to quantify the strength of these associations.

## 3. Result

In this study, 82 individuals were evaluated. The mean (±SD) age was 34.6 ± 4.6 years, BMI was 25.5 ± 5.21 kg/m^2^, gravidity was 3.7 ± 1.1, and parity was 2.3 ± 0.97. Gestational age ranged from 31 to 42 weeks, with a mean (±SD) of 35.9 ± 2.2 weeks, and the mean number of previous cesarean deliveries was 2 ± 0.8. The distribution of previous cesarean deliveries among participants revealed that cesarean repeat (R) III was the most common (40.2%), followed by R IV (31.7%).

The most common reason for the first cesarean section was failure to progress in normal vaginal delivery (NVD), accounting for 30.5% of cases, followed by fetal distress (18.3%). For the most recent cesarean section, the primary indication was a repeated cesarean section, representing 92.7% (76 out of 82 cases). Among these 76 repeated cesarean sections, the four most common causes were placenta accreta (23.7%, 18 cases), intrauterine growth restriction (IUGR) (10.5%, 8 cases), preeclampsia (6.6%, 5 cases), and rupture of membranes (ROM) (7.9%, 6 cases).

In our study, 56 patients (68.3%) were classified as ASA I, indicating that the majority of the patients were healthy without significant systemic disease. The demographic and obstetrical characteristics of the patients are summarized in Table 1.

As the primary outcome of the current study, placenta accreta (30.49%) was the most common cause of hysterectomy, followed by placenta previa (24.39%) and placenta increta (12.20%) (Table 2). Given the significant blood loss associated with these conditions, 50 out of 82 patients (60.98%) received blood transfusions. Among these, 79.3% were transfused with one or more units of plasma, with some patients receiving up to two units. Additionally, 22% received fresh frozen plasma (FFP), while only 3.7% were administered platelets.

In this study, inter-post-operative complications were observed in 54% of patients (45 out of 82 cases). The most common complications were bladder injury, occurring in 33.33% of cases (15 out of 45), followed by fever at 20% (9 out of 45), and ureteral injury at 13.33% (6 out of 45). The distribution of the causes of hysterectomy and inter-post-operative complications is presented in Table 2.

The secondary objective of the study was to examine the relationship between various factors and the complications associated with cesarean hysterectomy. A logistic regression analysis revealed that a higher BMI significantly increased the odds of complications (OR = 4.18, 95% CI: 1.66–10.51, *p* = 0.002), and the number of previous cesarean sections was also a significant predictor (OR = 2.30, 95% CI: 1.17–4.53, *p* = 0.016). However, variables such as age, gravidity, parity, causes of both the first and recent cesareans, as well as the causes leading to hysterectomy, did not show statistically significant associations with the outcome. In addition to these findings, among the 82 cases, there were 18 specific follow-up events recorded. These included 12 cases (14.63%) involving the removal of the Foley catheter within 2 weeks, six cases (7.32%) requiring the continuation of the Foley catheter and a subsequent visit to a urologist 3 weeks later, and one case (1.22%) necessitating a referral to a pulmonologist.

## 4. Discussion

The findings of our study provide important insights into the frequency, causes, and perioperative complications associated with cesarean hysterectomy. Over a 5-year period, 82 cases of cesarean hysterectomy were identified, with the most prevalent cause being placental abnormalities, particularly placenta accreta (30.49%). These results align with previous studies, which have identified placental adhesions as a leading cause of hysterectomy [6].

The increasing global prevalence of placenta accreta is a significant concern [3]. Recent data indicate that the estimated incidence of placenta accreta/increta/percreta is 1.7 per 10,000 maternities overall, rising to 577 per 10,000 in women with both a previous cesarean delivery and placenta previa [7]. This trend underscores the importance of early diagnosis and optimal management. The consistency of these findings highlights the critical role of placental abnormalities in contributing to the need for hysterectomy following cesarean delivery.

In our study, the distribution of causes further emphasized the prominence of placenta accreta and placenta previa, which were the most frequently observed contributors to cesarean hysterectomy. These findings are consistent with other recent studies [6,8]. For example, Veisi et al. reported that placental adhesions accounted for over half of all hysterectomies, with most patients having a history of prior cesareans [6]. Similarly, Forna et al. identified uterine atony and its association with hemorrhage as a significant cause of hysterectomy in their study [8]. These results suggest that both placental abnormalities and uterine atony are key factors driving the need for hysterectomy, with their prevalence varying depending on patient populations and surgical practices [8]. These results suggest that both placental abnormalities and uterine atony contribute to the need for hysterectomy, with varying prevalence depending on the patient population and surgical practices.

It is also important to compare these findings with cases of cesarean hysterectomy in patients who previously underwent fertility-sparing treatments for oncological reasons, such as breast or endometrial cancer, as these individuals may face unique risks and complications [9].

When examining secondary outcomes, we noted significant perioperative complications, with bladder injury occurring in 33.33% of cases in our study. The high incidence of bladder injuries—affecting one-third of cases—is striking and warrants further discussion. This finding aligns with studies highlighting the technical challenges and anatomical distortions associated with cesarean hysterectomy, particularly in cases involving placenta accreta spectrum disorders [10]. Consistent with this, a retrospective case series by Naicker et al. found that bladder injuries were the most common lower urinary tract injury (89.5%), primarily occurring during emergency cesarean deliveries, with previous cesarean sections and adhesions identified as significant risk factors [11]. Other complications, including fever, SSI, and ureteral injuries, were consistent with those reported in the literature [12,13]. SSI, a common complication after cesarean hysterectomy, increases hospital stays, morbidity, and costs, emphasizing the need for strict aseptic techniques and timely antibiotic prophylaxis [14]. Furthermore, rare complications such as utero-cutaneous fistula, though uncommon, should be considered in patients with persistent post-cesarean symptoms, as early diagnosis and management are crucial to preventing long-term morbidity [15].

These complications highlight the inherent challenges of performing cesarean hysterectomy, particularly in cases involving significant adhesions from previous cesarean deliveries. Careful surgical techniques, multidisciplinary collaboration, and thorough preoperative evaluation are essential to minimize the risk of damage to surrounding organs and reduce postoperative complications. Emerging research highlights Butyrylcholinesterase (BChE) as a potential predictive marker for SSI and septic complications [16].

An important finding of our study was the significant proportion of patients (60.95%) requiring blood transfusions, highlighting the invasive nature of cesarean hysterectomy and the associated risk of substantial blood loss. This aligns with previous studies reporting high transfusion rates in cesarean hysterectomy patients, underscoring the severity of intraoperative complications and the need for meticulous patient selection and management to mitigate risks [17]. Additionally, lower pre-delivery serum potassium (K+) levels have been identified as a potential risk factor for PPH, emphasizing the importance of monitoring electrolyte levels in high-risk patients to predict and prevent severe bleeding complications [18].

The results of the logistic regression analysis highlight the significant impact of a higher BMI and the number of previous cesarean sections on the likelihood of adverse outcomes in cesarean hysterectomy cases. BMI emerged as a robust predictor, with a fourfold increase in the odds of complications per unit rise in BMI. This finding aligns with the existing literature, where obesity is frequently associated with higher surgical risks due to increased operative time, technical difficulty, and a predisposition to complications such as infection and excessive bleeding [19,20,21]. These results underscore the importance of addressing obesity in preconception care to reduce surgical risks and improve outcomes.

Similarly, the number of previous cesarean sections was a significant predictor, with each additional cesarean nearly doubling the odds of complications. This is likely due to the cumulative risk of adhesions, uterine rupture, or abnormal placentation, which increase with each successive surgery. These findings highlight the importance of careful patient counseling and consideration of alternative delivery options in women with multiple prior cesarean deliveries to mitigate risks [22,23]. The findings also underscore the critical need for clinicians to weigh the risks of repeated cesareans and promote strategies to reduce their frequency, such as encouraging vaginal birth after cesarean (VBAC) when clinically appropriate. Interestingly, variables such as age, gravidity, parity, and causes of cesarean or hysterectomy did not show significant associations with the outcome. While these factors are often linked to maternal morbidity, their lack of significance in this study suggests that BMI and surgical history may be more decisive predictors. This could be attributed to the specific population studied or methodological variations, warranting further research in broader cohorts. Overall, these results highlight the importance of tailored interventions to address modifiable risk factors, such as BMI, and to carefully manage patients with a history of multiple cesareans to reduce adverse outcomes during cesarean hysterectomy.

In summary, the findings highlight the importance of preoperative assessment, particularly in identifying women with a history of multiple cesarean sections, as they are at higher risk of complications. This can guide surgical decision-making and planning. The significant odds ratios associated with BMI and the number of previous cesareans suggest these factors could serve as predictors to identify high-risk patients.

Limitations of this study include its retrospective design, which may introduce bias in data collection and analysis. Additionally, the single-center nature of the study may limit the generalizability of the results to other settings. Future research should focus on multi-center studies and prospective designs to further validate these findings.

## 5. Conclusions

In conclusion, this study provides valuable insights into the factors contributing to cesarean section-related hysterectomy, with placenta accreta identified as the leading cause. The analysis revealed a strong association between repeated cesarean deliveries and an increased risk of complications, particularly bladder and ureteral injuries. Logistic regression identified the number of previous cesarean sections and the frequency of cesarean repeats as significant predictors of adverse outcomes. The study also highlights the importance of careful management and follow-up in patients undergoing cesarean hysterectomy, as a notable proportion required additional medical interventions. These findings underscore the need for enhanced preoperative assessment and surgical precision to mitigate complications and improve patient outcomes.

## Figures and Tables

**Table 1:** Demographic and obstetrical characteristics of patients (n = 82).

Variable	Min–Max	Mean (SD)
Age, y	23–48	34.6 (4.6)
BMI, kg/m^2^	22.4–40.5	25.5 (5.21)
Gravidity, n	2–7	3.7 (1.1)
Parity, n	1–5	2.3 (0.97)
Abortion, n	0–5	0.5 (0.8)
Number of previous CDs, n	1–5	2 (0.8)
GA, w	31–42	35.9 (2.2)
Indication of first CD, n (%)	Macrosomia	4 (4.88)
	Preeclampsia	3 (3.66)
	Breech presentation	10 (12.20)
	Transverse lie	2 (2.44)
	Fetal distress	15 (18.29)
	Failure to progress	25 (30.49)
	Decreased FM	3 (3.66)
	Decreased AF	8 (9.76)
	Meconium-stained	12 (14.63)
Indication of latest CD, n (%)	Repeated cesarean	76 (92.68)
	Failure to progress	1 (1.22)
	Preeclampsia	2 (2.44)
	Elective caesarean section	1 (1.22)
	Breech presentation	1 (1.22)
	Placenta Previa	1 (1.22)
ASA score	ASA I	56 (68.3)
	ASA II	18 (22)
	ASA III	8 (9.7)

Abbreviations: AF: Amniotic Fluid; ASA: American Society of Anesthesiologists; BMI: Body Mass Index; CD: Cesarean Delivery; FM: Fetal Movement; GA: Gestational Age.

**Table 2:** Distribution of cause of hysterectomy and perioperative complications.

Variable		Number	Percentage
Cause of hysterectomy	Uterine atony	6	7.32
	Placenta accreta	25	30.49
	Placenta previa	20	24.39
	Placenta increta	10	12.20
	Placenta percreta	13	15.85
	Uterine rupture	2	2.44
Complications *	Bladder injury	15	33.33
	Bowel injury	2	4.44
	Fever	9	20
	Hematoma	4	8.89
	Surgical site infections	5	11.1
	Abdominal reoperation	3	6.67
	Pulmonary embolism	1	2.22
	Ureteral injury	6	13.33
	Fistula	0	0

* Complications were observed in 45 out of 82 cases, and the percentage of each complication was calculated based on these 45 cases.

## Data Availability

The raw data supporting the conclusions of this article will be made available by the authors on request.
